# PGAM5-Mediated PHB2 Dephosphorylation Contributes to Diabetic Cardiomyopathy by Disrupting Mitochondrial Quality Surveillance

**DOI:** 10.34133/research.0001

**Published:** 2022-12-15

**Authors:** Rongjun Zou, Jun Tao, Jie He, Chaojie Wang, Songtao Tan, Yu Xia, Xing Chang, Ruibing Li, Ge Wang, Hao Zhou, Xiaoping Fan

**Affiliations:** ^1^ Department of Cardiovascular Surgery, Guangdong Provincial Hospital of Chinese Medicine, The Second Affiliated Hospital of Guangzhou University of Chinese Medicine, Guangzhou 510120, Guangdong, China.; ^2^ The Second Clinical College of Guangzhou University of Chinese Medicine, Guangzhou 510405, Guangdong, China.; ^3^ Department of Cardiovascular Surgery, Sun Yat-sen Memorial Hospital, Sun Yat-sen University, Guangzhou 510120, Guangdong, China.; ^4^ Senior Department of Cardiology, The Sixth Medical Center of People’s Liberation Army General Hospital, Beijing 100048, China.; ^5^ Guang’anmen Hospital, China Academy of Chinese Medical Sciences, Beijing 100053, China.

## Abstract

Disruption of the mitochondrial quality surveillance (MQS) system contributes to mitochondrial dysfunction in diabetic cardiomyopathy (DCM). In this study, we observed that cardiac expression of phosphoglycerate mutase 5 (PGAM5), a mitochondrial Ser/Thr protein phosphatase, is upregulated in mice with streptozotocin-induced DCM. Notably, DCM-related cardiac structural and functional deficits were negated in cardiomyocyte-specific *Pgam5* knockout (*Pgam5^CKO^*) mice. Hyperglycemic stress impaired adenosine triphosphate production, reduced respiratory activity, and prolonged mitochondrial permeability transition pore opening in acutely isolated neonatal cardiomyocytes from control *Pgam5^f/f^* mice, and these effects were markedly prevented in cardiomyocytes from *Pgam5^CKO^* mice. Likewise, three main MQS-governed processes—namely, mitochondrial fission/fusion cycling, mitophagy, and biogenesis—were disrupted by hyperglycemia in *Pgam5^f/f^*, but not in *Pgam5^CKO^*, cardiomyocytes. On the basis of bioinformatics prediction of interaction between PGAM5 and prohibitin 2 (PHB2), an inner mitochondrial membrane-associated scaffolding protein, co-immunoprecipitation, and immunoblot assays demonstrated that PGAM5 dephosphorylates PHB2 on Ser91. Transfection of cardiomyocytes with phosphodefective or phosphomimetic Ser91 mutants of PHB2 confirmed a critical role for PGAM5-mediated dephosphorylation of PHB2 in mitochondrial dysfunction associated with hyperglycemic stress. Furthermore, knockin mice expressing phosphomimetic PHB2^S91D^ were resistant to diabetes-induced cardiac dysfunction. Our findings highlight the PGAM-PHB2 axis as a novel and critical regulator of mitochondrial dysfunction in DCM.

## Introduction

Diabetic cardiomyopathy (DCM), a major complication of type 2 diabetes, is a pathological condition featured by cardiac hypertrophy, myocardial fibrosis, abnormal microvascular perfusion, and decreased heart function [1]. Numerous molecular mechanisms contributing to reduced performance of the diabetic heart have been reported [2]. Mitochondrial dysfunction has been observed in the heart tissue from diabetic patients and is regarded as a main pathogenic basis during DCM progression [3]. Indeed, molecular studies have linked mitochondrial metabolic disorder [4], increased mitochondrial network fragmentation [5], mitochondrial calcium mishandling [6], mitochondrial oxidative stress [7], and proteomic remodeling and mitochondrial DNA damage [8] with DCM. In response to mitochondrial dysfunction, the mitochondrial quality surveillance (MQS) system becomes activated to remove or repair damaged mitochondria and restore mitochondrial homeostasis. Although the mitochondrial processes governed by the MQS (fission/fusion cycles, mitophagy, and biogenesis) have been explored in-depth by studies by others [5,9] and us [10,11], upstream regulatory mechanisms that control MQS function in the setting of DCM are still incompletely defined.

Phosphoglycerate mutase 5 (PGAM5) is a mitochondrial Ser/Thr phosphatase mainly expressed in the inner mitochondrial membrane (IMM). Early studies reported that PGAM5 is markedly elevated in response to cardiac stress resulting from myocardial ischemia–reperfusion injury [12], chronic doxorubicin exposure [13], and lipopolysaccharide exposure [14]. Pharmacological inhibition or genetic ablation of PGAM5 correlates with improved heart function in response to stress [15,16]. Repression of PGAM5 inhibits Drp1-dependent mitochondrial fission [12], blocks the mitochondrion-related apoptosis pathway [15], and interrupts mitochondrial permeability transition pore (mPTP)-mediated necroptosis [17], highlighting that PGAM5 functions as a pivotal effector for the induction of mitochondrial dysfunction in various cardiovascular disorders. This concept is further validated by our recent study [18], which showed that cardiac-specific *Pgam5* knockout (*Pgam5*-KO; *Pgam5^CKO^*) mice are less vulnerable to myocardial ischemia–reperfusion injury due to improved MQS. Importantly, global *Pgam5*-KO mice were found to be highly resistant to high-fat diet-related obesity, in association with reductions in serum triglycerides and lipid content in brown adipose tissue (BAT) and enhanced fibroblast growth factor 21 (FGF21) paracrine signaling [19]. In turn, increased levels of uncoupling protein 1 (UCP1) and enhanced oxygen consumption rate have been demonstrated in brown adipocytes from *Pgam5*-KO mice [20]. These observations suggest that PGAM5 may be involved into the regulation of metabolic diseases.

Prohibitin 2 (PHB2) acts as mitochondrial scaffolding/chaperone protein that assembles at the IMM to form a ring-like structure that maintains distinctive mitochondrial functions, especially MQS [21]. Early studies showed that PHB2 deficiency suppresses pancreatic β-cell function and, thus, promotes diabetes progression through inducing mitochondrial fragmentation [22]. Moreover, experiments in mice revealed that cardiac PHB2 deficiency is associated with increased formation of lipid droplets, impaired fatty acid oxidation due to decreased mitochondrial function, and increased susceptibility to heart failure [23]. Of note, recent studies further illustrated the functional importance of posttranslational PHB2 phosphorylation in regulating MQS. For instance, it was shown that PHB2 phosphorylation at Ser91 promotes human leukemia cell progression through repressing mitochondrial apoptosis [24], whereas phosphorylation at Ser39 mediates PHB2 association with AURKA and MAP1LC3 to elicit Parkin-independent mitophagy [25].

In light of the evidence connecting PGAM5 and PHB2 with mitochondrial dysfunction and metabolic anomalies commonly observed in DCM, as well as the bioinformatics analysis predicting a potential interaction between these proteins, we tested the hypothesis that PGAM5-mediated dephosphorylation of PHB2 disrupts MQS to aggravate myocardial dysfunction in DCM.

## Results

### Cardiac expression of PGAM5 is upregulated in diabetic mice

**Fig. 1.**
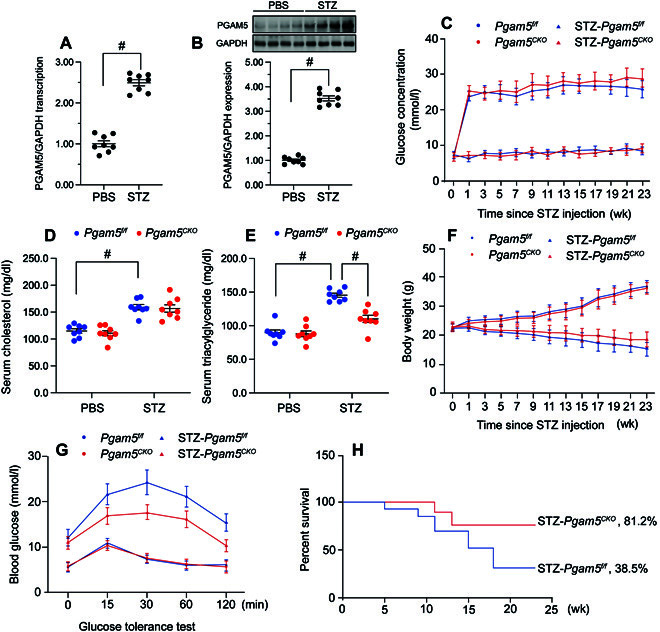
Cardiac PGAM5 expression is upregulated by hyperglycemia and contributes to metabolic disorder. In vivo, the cardiomyocyte-specific *Pgam5* knockout (*Pgam5^CKO^*) and *Pgam5^f/f^* mice were injected intraperitoneally with STZ (50 mg/kg dissolved in 0.1 mol/l of citrate buffer) for 5 consecutive days to induce diabetes. Nondiabetic mice were the age- and sex-matched, which were injected with the same volume of PBS. (A) Analysis of relative transcription levels of *Pgam5* in cardiac tissues by qPCR. GAPDH, glyceraldehyde phosphate dehydrogenase. (B) Western blot analysis of PGAM5 expression in mice hearts. (C) Time course for changes in fasting blood glucose levels. Blood glucose was measured at baseline (week 0) and periodically over the course of 24 weeks after the first day of STZ or citrate buffer (vehicle control) injection. (D and E) Determination of fasting serum cholesterol (D) and triglyceride (E) levels in mice, 24 weeks after STZ or vehicle injection. (F) Time course of body weight changes. (G) IPGTT results for *Pgam5^CKO^* and *Pgam5^f/f^* mice. Tests were performed 9 days after STZ/vehicle treatment. (H) Kaplan-Meier survival curves for the various groups (*n* = 24 mice). Values are presented as mean ± SEM. For biochemical determinations, *n* = 6 mice per group. #*P* < 0.05.

Diabetes-induced alterations in cardiac PGAM5 expression were explored in mice injected with streptozotocin (STZ). Expression analyses by quantitative polymerase chain reaction (qPCR) (Fig. 1A) and western blotting (Fig. 1B) showed that PGAM5 mRNA and protein levels in heart tissue were markedly elevated 1 month after STZ treatment. To evaluate whether PGAM5 overexpression contributes to DCM, we generated cardiomyocyte-specific *Pgam5*-KO (*Pgam5^CKO^*) mice. Baseline metabolic parameters, including fasting plasma glucose (Fig. 1C), serum cholesterol (Fig. 1D), serum triglycerides (Fig. 1E), body weight (Fig. 1F), and glucose tolerance (Fig. 1G), did not differ between *Pgam5^CKO^* and *Pgam5^f/f^* control mice. Following STZ administration, increased fasting plasma glucose, serum cholesterol, and serum triglyceride levels indicated diabetes induction in both genotypes (Fig. 1C to G). There were no marked differences in fasting glucose levels (Fig. 1C), serum cholesterol (Fig. 1D), and body weight (Fig. 1F) between diabetic *Pgam5^f/f^* and *Pgam5^CKO^* mice. However, serum triglyceride was mildly reduced in *Pgam5^CKO^* mice compared to *Pgam5^f/f^* mice (Fig. 1E). Of note, after diabetes induction, *Pgam5^CKO^* mice showed a prolonged survival rate compared to *Pgam5^f/f^* mice (Fig. 1H).

### PGAM5 deficiency prevents myocardial dysfunction in diabetic mice

**Fig. 2.**
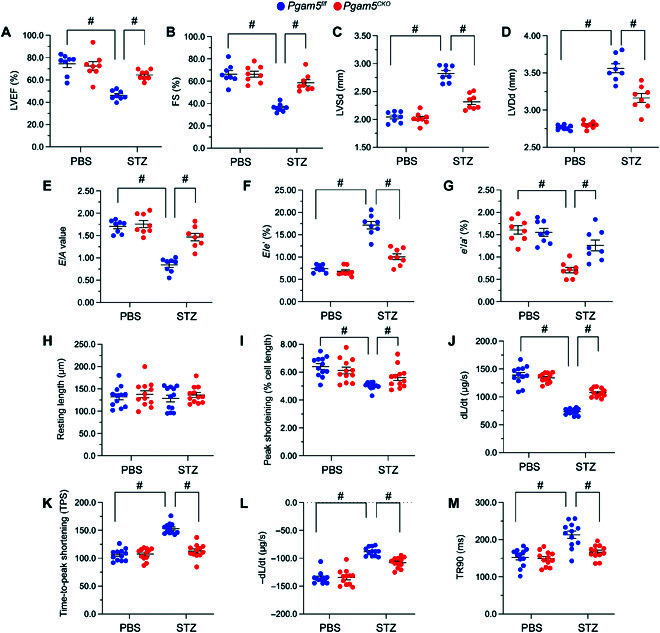
PGAM5 deficiency sustains myocardial function in diabetic mice. In vivo, the *Pgam5^CKO^* and *Pgam5^f/f^* mice were injected intraperitoneally with STZ (50 mg/kg dissolved in 0.1 mol/l of citrate buffer) for 5 consecutive days to induce diabetes. Nondiabetic mice were the age- and sex-matched, which were injected with the same volume of PBS. In vitro, cardiomyocytes were isolated from STZ-treated *Pgam5^CKO^* and *Pgam5^f/f^* mice. (A to G) Echocardiography analysis. (A) LVEF, left ventricular ejection fraction; (B) FS, fractional shortening; (C) LVSd, left ventricular systolic dimension; (D) LVDd, left ventricular diastolic dimension; (E) *E*/*A*, early to late (atrial) mitral flow velocity ratio; (F) *E*/*e*′, ratio of mitral peak velocity of early filling to early diastolic mitral annular velocity; and (G) *e*′/*a*′, ratio of diastolic mitral annulus velocities. (H to M) Analysis of contractile parameters in acutely isolated, single cardiomyocytes from *Pgam5^CKO^* and *Pgam5^f/f^* mice. (H) Resting length in cardiomyocytes; (I) PS, peaking shortening; (J) +dL/dt, maximal velocity of shortening; (K) TPS, time-to-peak shortening; (L) −dL/dt, maximal velocity of relengthening; and (M) TR90, time-to-90% relengthening. Values are presented as mean ± SEM. For in vivo data, *n* = 6 mice per group. For in vitro experiments, *n* = 4 independent experiments. #*P* < 0.05.

Next, echocardiography was used to evaluate the outcome of PGAM5 deletion on cardiac function in diabetic mice (Fig. 2A to G). In *Pgam5^f/f^* mice, STZ treatment impaired cardiac systolic capacity, evidenced by decreased left ventricular ejection fraction, reduced fractional shortening, and increased left ventricular systolic dimension (Fig. 2A to C). Likewise, myocardial diastolic indexes, such as ratio of early to late transmitral flow velocities (*E*/*A*), ratio of diastolic mitral annulus velocities (*e*′/*a*′), ratio of mitral peak velocity of early filling to early diastolic mitral annular velocity (*E*/*e*′), and left ventricular diastolic dimension, were also impaired after STZ injection in *Pgam5^f/f^* mice (Fig. 2D to G). However, these alterations were negated in diabetic *Pgam5^CKO^* mice (Fig. 2A to G).

Subsequently, contractile parameters were analyzed in freshly isolated, single cardiomyocytes from nondiabetic and diabetic mice. STZ administration had no impact on resting length in cardiomyocytes from either *Pgam5^f/f^* or *Pgam5^CKO^* mice (Fig. 2H). However, upon electrical field stimulation, impaired contractility, evidenced by reductions in peak shortening, maximal velocity of shortening, and time-to-peak shortening, were recorded in cardiomyocytes isolated from STZ-treated *Pgam5^f/f^* mice (Fig. 2I to K). These cells showed also abnormal relaxation kinetics, reflected by reductions in maximal velocity of relengthening and time-to-90% relengthening (Fig. 2L and M). In contrast, contractile/relaxation properties were improved in cardiomyocytes from STZ-treated *Pgam5^CKO^* mice (Fig. 2I to M). These results suggest that PGAM5 promotes cardiac dysfunction in STZ-induced DCM.

### PGAM5 deletion prevents DCM-related myocardial structural disorder

**Fig. 3.**
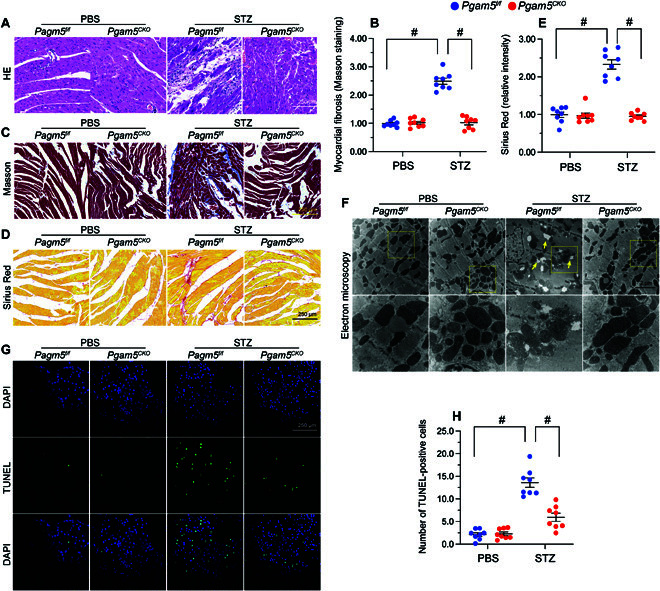
PGAM5 deletion prevents diabetes-mediated myocardial structural disorder. In vivo, the *Pgam5^CKO^* and *Pgam5^f/f^* mice were injected intraperitoneally with STZ (50 mg/kg dissolved in 0.1 mol/l of citrate buffer) for 5 consecutive days to induce diabetes. Nondiabetic mice were the age- and sex-matched, which were injected with the same volume of PBS. (A) Representative histopathological images (H&E staining) depicting myocardial disarray in diabetic mice. (B) Representative histopathological images (Masson trichrome staining) revealing myocardial fibrosis in diabetic mice. (C) Quantification of Masson trichrome staining. (D) Representative histopathological images (Sirius Red staining) revealing myocardial fibrosis in diabetic mice. (E) Quantification of Sirius Red staining. (F) Representative electron microscopy images depicting ultrastructural changes in heart tissue from diabetic mice. Yellow arrows indicate disordered myofibrils and swollen mitochondria. (G) Representative images of TUNEL staining in heart tissues from vehicle- and STZ-treated mice and corresponding quantification data. DAPI, 4′,6-diamidino-2-phenylindole. (H) Quantification of TUNEL staining. Values are presented as mean ± SEM; *n* = 6 mice per group. #*P* < 0.05.

Following STZ treatment, myocardial disarray, as assessed by H&E staining (Fig. 3A), and myocardial fibrosis, revealed by Masson trichrome/Sirius Red stain (Fig. 3B to E), were detected in *Pgam5^f/f^* mice but not in *Pgam5^CKO^* mice. Western blot analysis further displayed that STZ injection markedly promoted collagen I/III accumulation in heart tissue from *Pgam5^f/f^* mice, but not *Pgam5^CKO^* mice (Fig. S1A to E). In addition, cardiac transforming growth factor-β (TGFβ) and matrix metallopeptidase 9 (MMP9) levels were increased in *Pgam5^f/f^* mice but showed near-normal levels in *Pgam5^CKO^* mice (Fig. S1A to E). Ultrastructural changes in the myocardium were subsequently analyzed by electron microscopy. STZ treatment was associated with disordered myofibrils, swollen mitochondria, and reduced mitochondrial number in heart samples from *Pgam5^f/f^* mice (Fig. 3F). However, loss of *Pgam5* in cardiomyocytes largely prevented myocardial ultrastructural changes induced by STZ (Fig. 3F). Moreover, terminal deoxynucleotidyl transferase–mediated deoxyuridine triphosphate nick end labeling (TUNEL) staining showed induction of cardiomyocyte apoptosis in STZ-treated *Pgam5^f/f^* mice, and this effect was markedly reduced upon deletion of *Pgam5* (Fig. 3C and H). These findings strongly indicate that PGAM5 deficiency prevents deleterious changes in myocardial structure associated with DCM.

### PGAM5 ablation attenuates hyperglycemia-mediated mitochondrial damage

**Fig. 4.**
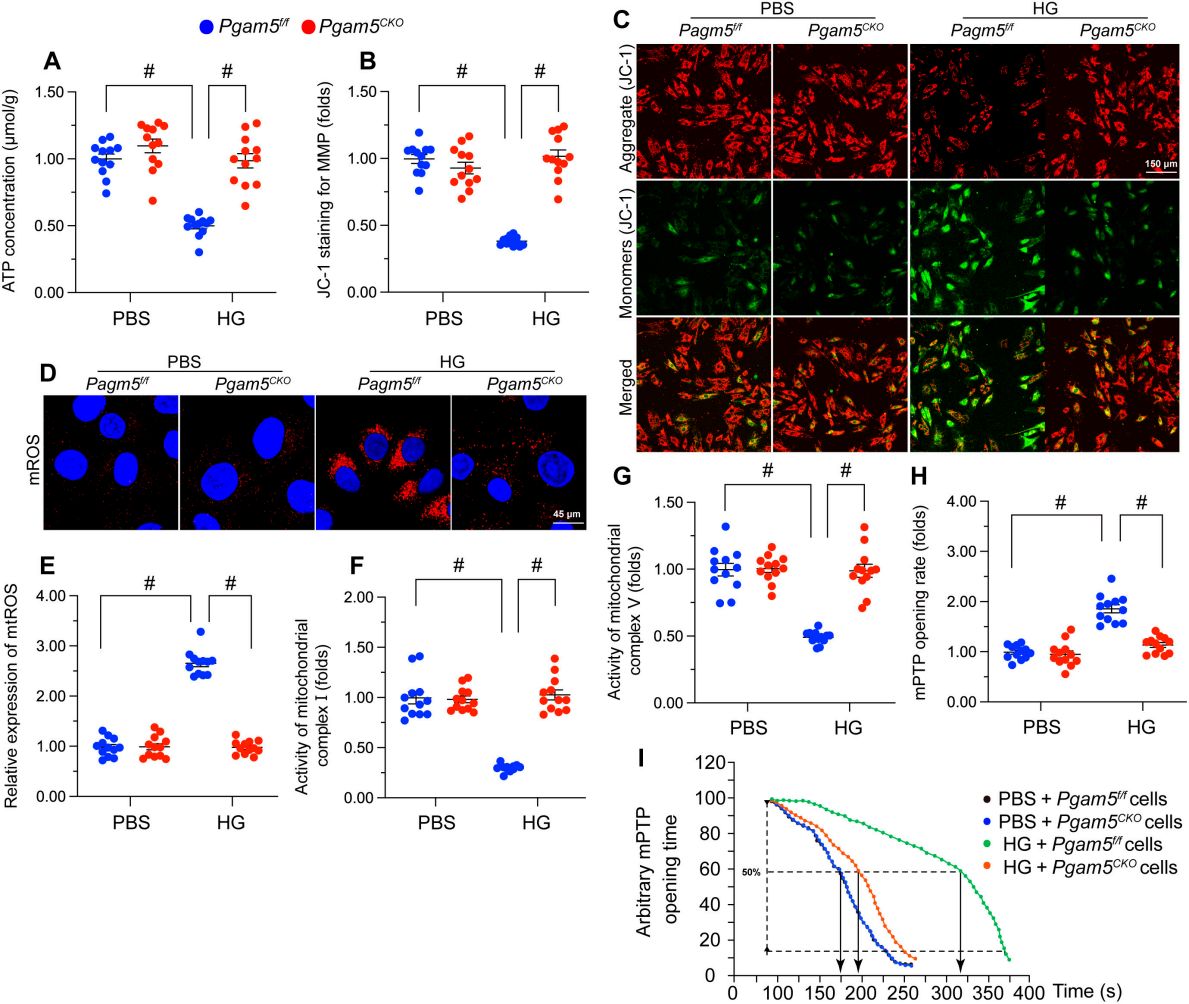
PGAM5 ablation attenuates hyperglycemia-mediated mitochondrial damage. Primary neonatal cardiomyocytes isolated from *Pgam5^CKO^* and *Pgam5^f/f^* mice were cultured under high glucose (30 mmol/l) medium for 48 h to induce hyperglycemic stress in vitro. Cardiomyocytes incubated in normal glucose (5.5 mmol/l) medium were used as control. (A) ELISA-based analysis of ATP production in primary cardiomyocytes. (B) Detection of mitochondrial membrane potential (ΔΨm) in cardiomyocytes loaded with the JC-1 probe. (C) The red-to-green fluorescence ratio was used for semiquantitative analysis of changes in ΔΨm. (D) Analysis of mtROS generation in cardiomyocytes loaded with MitoSOX Red. (E) The levels of mtROS were normalized against those of the control group. (F and G) ELISA-based determination of the activity of mitochondrial respiratory complex I (F) and complex V (G). (H) Detection of mPTP opening in cardiomyocytes loaded with TMRE. (I) Quantification of mPTP in cardiomyocytes. Values are presented as mean ± SEM from 4 independent experiments. #*P* < 0.05.

To describe the influence of PGAM5 on cardiac mitochondrial homeostasis during DCM, primary neonatal cardiomyocytes isolated from *Pgam5^f/f^* and *Pgam5^CKO^* mice were incubated with high-glucose (30 mmol/l) medium. Cells incubated in normal glucose (5.5 mmol/l) medium were used as controls. After exposure to hyperglycemic media, adenosine triphosphate (ATP) production was markedly reduced in *Pgam5^f/f^* but not in *Pgam5^CKO^* cardiomyocytes (Fig. 4A). Since ATP production is critically influenced by mitochondrial membrane potential (ΔΨm), the JC-1 probe was used to analyze this parameter. Hyperglycemic stress dissipated ΔΨm in *Pgam5^f/f^* cardiomyocytes (Fig. 4B and C) and elevated mitochondrial reactive oxygen species (mtROS) generation (Fig. 4D and E), as assessed by the fluorescent superoxide marker MitoSOX Red. However, under hyperglycemic conditions, deletion of *Pgam5* in cardiomyocytes not only preserved ΔΨm (Fig. 4B and C) but also repressed also mtROS generation (Fig. 4D and E). Since ATP and mtROS production are generated by the mitochondrial respiration complex, enzyme-linked immunosorbent assay (ELISA) was applied to analyze the activity of mitochondrial respiration complex in both control and hyperglycemic cells. Results showed that hyperglycemia impaired the activity of complexes I and V in *Pgam5^f/f^* cardiomyocytes, and this effect was attenuated in *Pgam5^CKO^* cells (Fig. 4F and G). Because mitochondrial damage triggers cellular apoptosis through prolonged opening of the mPTP, this parameter was next assessed by tetramethylrhodamine ethyl ester (TMRE) fluorescence (Fig. 4H and I). Hyperglycemia extended the mPTP opening time in *Pgam5^f/f^* cardiomyocytes, whereas this change was negated in *Pgam5^CKO^* cardiomyocytes (Fig. 4H and I). These results indicate that PGAM5 deletion markedly attenuates hyperglycemia-mediated mitochondrial dysfunction in cardiomyocytes.

### PGAM5 deletion sustains MQS in hyperglycemia-challenged cardiomyocytes

**Fig. 5.**
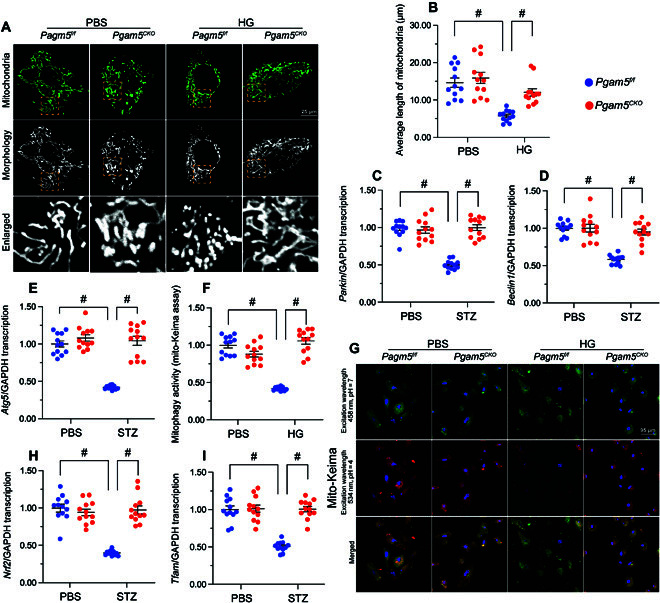
PGAM5 deletion normalizes MQS in cardiomyocytes exposed to hyperglycemia. In vivo, the *Pgam5^CKO^* and *Pgam5^f/f^* mice were injected intraperitoneally with STZ (50 mg/kg dissolved in 0.1 mol/l of citrate buffer) for 5 consecutive days to induce diabetes. Nondiabetic mice were the age- and sex-matched, which were injected with the same volume of PBS. Primary neonatal cardiomyocytes isolated from *Pgam5^CKO^* and *Pgam5^f/f^* mice were cultured under high-glucose (30 mmol/l) medium for 48 h to induce hyperglycemic stress in vitro. Cardiomyocytes incubated in normal glucose (5.5 mmol/l) medium were used as control. (A) Tom20 immunofluorescence was applied for analysis of hyperglycemia-induced alterations of mitochondrial morphology in cardiomyocytes. (B) Mean mitochondrial length was analyzed in at least 100 cardiomyocytes. (C to E) qPCR analysis of *Parkin* (C), *Beclin1* (D), and *Atg5* (E) expression in cardiac tissue. (F) Analysis of mitophagy in primary cardiomyocytes expressing the mito-Keima probe. (G) Presentative pictures of cardiomyocyte transfected with mito-Keima. (H and I) Relative transcription levels of *Nrf2* (H) and *Tfam* (I) in cardiac tissue, assessed by qPCR. Values are presented as mean ± SEM. For in vivo data, *n* = 6 mice per group. For in vitro data, *n* = 4 independent experiments. #*P* < 0.05.

We next asked whether PGAM5 expression contributes to mitochondrial dysfunction in DCM by disrupting the MQS system, which maintains cellular energy homeostasis by regulating mitochondrial fusion and fission cycles, mitophagy, and mitochondrial biogenesis. In heart samples from STZ-treated *Pgam5^f/f^* mice, qPCR analysis of mitochondrial dynamics-related genes showed that the levels of *Drp1* and *Fis1* were increased while the expression of *Mfn2* and *Opa1* were instead suppressed (Fig. S2A to D). These findings suggested that mitochondrial fission is enhanced, whereas fusion is suppressed, in cardiac tissue subjected to hyperglycemic stress. In contrast, normalized levels of *Drp1*/*Fis1* and *Mfn2*/*Opa1* were observed in heart tissues from STZ-treated *Pgam5^CKO^* mice (Fig. S2A to D). Consistent with these in vivo changes, Tom20 immunofluorescence (applied to assess mitochondrial morphology) displayed a reduction in the average mitochondrial length and an increase in the ratio of fragmented to tubular mitochondria in hyperglycemia-treated *Pgam5^f/f^* cardiomyocytes (Fig. 5A and B). In turn, normalized mitochondrial morphology, evidenced by preserved mitochondrial length and decreased number of fragmented mitochondria, was observed under hyperglycemic conditions in *Pgam5*-deficient cardiomyocytes (Fig. 5A and B). These results revealed that disruption of mitochondrial dynamics caused by hyperglycemia can be rectified by PGAM5 deletion.

Mitophagy is primarily activated by Parkin and other autophagy-related proteins such as Beclin1 and ATG5. Compared to nondiabetic *Pgam5^f/f^* mice, STZ-treated *Pgam5^f/f^* mice showed decreased levels of *Parkin*, *Beclin1*, and *Atg5* (Fig. 5C to E). However, these changes were abrogated in STZ-treated *Pgam5^CKO^* mice (Fig. 5C to E). Supporting these observations, mitophagy analysis using the mito-Keima probe further showed that hyperglycemia reduced mitophagy in *Pgam5^f/f^* cardiomyocytes but not in *Pgam5^CKO^* cardiomyocytes (Fig. 5F and G). These findings indicate that PGAM5 deficiency rescues mitophagy in hyperglycemia-exposed cardiomyocytes.

Mitochondrial biogenesis is controlled by peroxisome proliferator-activated receptor-gamma coactivator-1alpha (PGC-1α) and its downstream transcriptional coactivators *Nrf2* and *Tfam*. The qPCR illuminated that the mRNA expression of *Pgc1α*, *Nrf2*, and *Tfam* was inhibited in STZ-treated *Pgam5^f/f^* mice compared with nondiabetic *Pgam5^f/f^* mice (Fig. 5H and I and Fig. S2E). In contrast, and suggesting a critical role for PGAM5 in hyperglycemia-mediated suppression of mitochondrial biogenesis, these expression changes were nullified in STZ-treated *Pgam5^CKO^* mice (Fig. 5H and I and Fig. S2E).

### PGAM5 binds and dephosphorylates PHB2

**Fig. 6.**
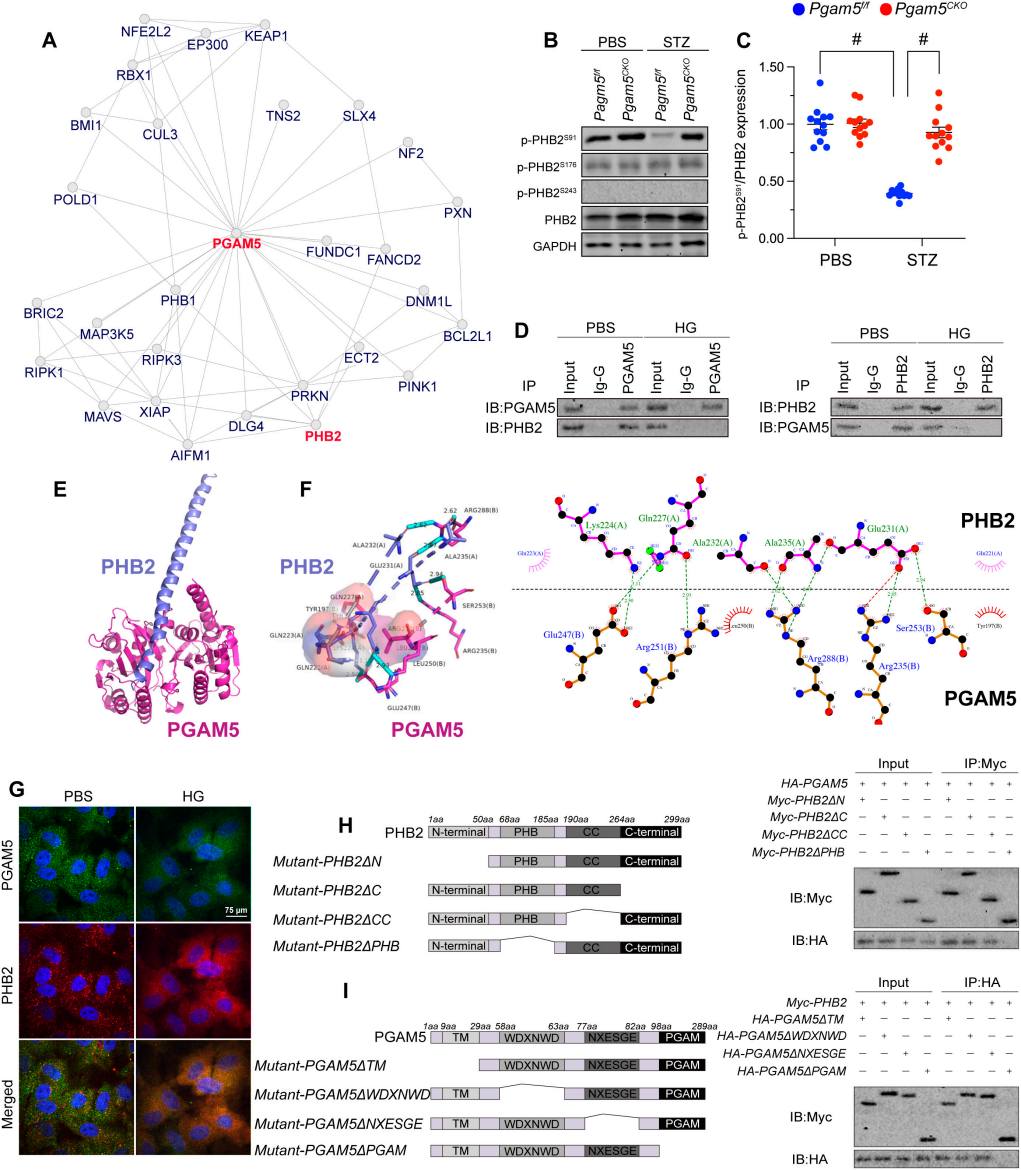
PGAM5 binds and dephosphorylates PHB2. Mouse HL-1 cardiomyocyte cell line was cultured under high-glucose medium (30 mmol/l of glucose) for 48 h to induce hyperglycemia damage in vitro. HL-1 cells treated with normal glucose medium (5.5 mmol/l of glucose) was used as the control group. (A) Potential PGAM5-PHB2 interaction, as predicted in the inBio Discover platform. (B) Western blot analysis of p-PHB2^S91^ expression in cardiac tissues. (C) Western blot signals were normalized against total PHB2 expression. (D) Co-IP assay results indicating interaction between PGAM5 and PHB2. Ig-G, immunoglobulin G. (E) Molecular docking of PHB2 and PGAM5. (F) The potential binding sites between PHB2 and PGAM5 are shown in different colors. (G) Double immunofluorescence of PGAM5 and PHB2 in HL-1 cardiomyocytes exposed to hyperglycemia. (H) Mapping of regions in PHB2. Immunoprecipitation (IP) and immunoblotting (IB) were applied to assess interactions between PHB2 mutants and PGAM5. (I) Mapping of regions in PGAM5. Immunoprecipitation and immunoblotting were applied to assess interactions between region-specific PHB2 and PGAM5 mutants upon transfection into HL-1 cells. Values are presented as mean ± SEM. For in vivo data, *n* = 6 mice per group. For in vitro data, *n* = 4 independent experiments. #*P* < 0.05.

To address the mechanism by which PGAM5 impairs MQS in DCM, we focused on potential alterations in PHB2, an IMM-associated scaffolding protein reported to be a key regulator of mitophagy and MQS. Both qPCR (Fig. S3A) and western blotting (Fig. S3B) showed that baseline cardiac PHB2 expression was not affected by either diabetes induction or cardiac-specific *Pgam5* KO. However, on the basis of predicted interaction between PGAM5 and PHB2, revealed by the inBio Discover platform (https://inbio-discover.com) (Fig. 6A), we speculated that PGAM5 might affect PHB2 function through posttranscriptional modification. Considering that PGAM5 is a Ser/Thr phosphatase, and in light of evidence indicating that PHB2 phosphorylation improves MQS [24,25], we asked whether PGAM5 mediates PHB2 dephosphorylation to compromise MQS in DCM. PHB2 has at least 7 potential phosphorylation sites [26], of which only 3 (S91, S176, and S243) have been biochemically validated [24,27–31]. After STZ administration, western blot analyses on heart tissues showed reduced PHB2 phosphorylation, mainly at S91, in *Pgam5^f/f^* mice (Fig. 6B and C). However, this alteration was not observed in *Pgam5^CKO^* mice (Fig. 6B and C). Co-immunoprecipitation (Co-IP) assay further verified that the interaction between PGAM5 and PHB2 was induced upon hyperglycemia treatment (Fig. 6D and E). Moreover, the molecular docking assay in Fig. 6F to H showed the potential interactive sites between PHB2 and PGAM5. Immunofluorescence showed that, in the presence of hyperglycemia, the interaction between PGAM5 and PHB2 was enhanced relative to the baseline (Fig. 6I).

To define the specific regions involved in the interaction between PGAM5 and PHB2, the expression of four PHB2 and four PGAM5 deletion mutants was induced by transfection of the corresponding constructs into HL-1 cardiomyocytes (Fig. 6J and K). Co-IP assays showed that the interaction between PHB2 and PGAM5 was disrupted by a PHB2 mutant lacking the PHB domain (PHB2ΔPHB) (Fig. 6J) or by a PGAM5 mutant lacking the PGAM domain (PGAM5ΔPGAM) (Fig. 6K).

To confirm whether the interaction between PGAM5 and PHB2 is necessary for hyperglycemia-mediated PHB2 dephosphorylation, PGAM5 or PHB2 mutants were transfected into HL-1 cardiomyocytes prior to exposure to high glucose. As expected, hyperglycemia inhibited the levels of p-PHB2^S91^, and this effect was abolished by PGAM5ΔPGAM transfection (Fig. S4A and B). In turn, hyperglycemia-mediated PHB2 dephosphorylation at S91 was not affected by PHB2 mutants lacking the N-terminal domain (PHB2ΔN), the C-terminal domain (PHB2ΔC), or the coiled-coil domain (PHB2ΔCC) but was instead prevented by mutants lacking the PHB domain (PHB2ΔPHB) (Fig. S4A and B). These findings show that, upon hyperglycemia-mediated upregulation, PGAM5 directly binds and dephosphorylates PHB2 at S91.

### PGAM5 deficiency-mediated mitochondrial protection is abolished by transfection of a phosphorylation-defective PHB2 mutant

**Fig. 7.**
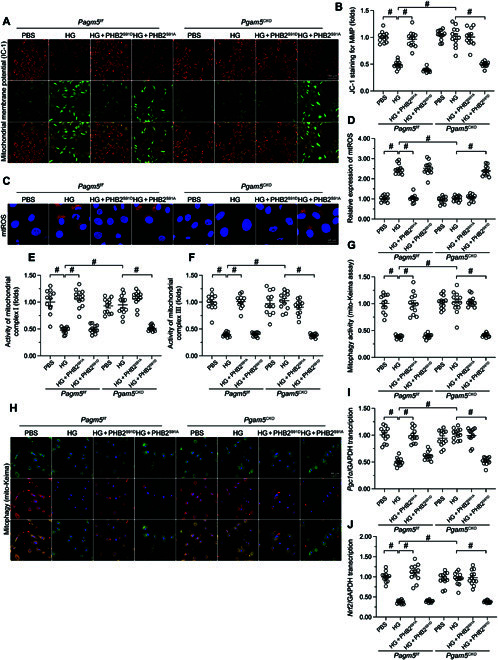
PGAM5 deficiency-mediated mitochondrial protection is abolished by transfection of a phosphorylation-defective PHB2 mutant. Primary neonatal cardiomyocytes were isolated from *Pgam5^CKO^* and *Pgam5^f/f^* mice, transfected with HA-PHB2^S91A^ or HA-PHB2^S91D^ mutant constructs, and exposed to PBS or hyperglycemia. (A) Detection of ΔΨm in cardiomyocytes loaded with the JC-1 probe. (B) The red-to-green fluorescence ratio was used for the semiquantitative analysis of changes in ΔΨm. (C) Analysis of mtROS generation in cardiomyocytes loaded with MitoSOX Red. (D) The levels of mtROS were normalized to those of the control group. (E and F) ELISA-based determination of the activity of mitochondrial respiratory complex I (E) and complex V (F). (G) Analysis of mitophagy in primary cardiomyocytes expressing the mito-Keima probe. (H) Presentative pictures of cardiomyocyte transfected with mito-Keima. (I and J) Analysis of *Pgc1α* (I) and *Nrf2* (J) transcription levels in cultured cardiomyocytes. Values are presented as mean ± SEM from 4 independent experiments. #*P* < 0.05.

To confirm whether normalization of mitochondrial homeostasis conferred by PGAM5 deficiency in hyperglycemic conditions is attributable to PHB2 dephosphorylation, we transfected *Pgam5^CKO^* cardiomyocytes with phosphodefective (HA-PHB2^S91A^) or phosphomimetic (HA-PHB2^S91D^) PHB2 constructs. Following exposure to hyperglycemia, the stabilizing effects of *Pgam5* deletion on ΔΨm (Fig. 7A and B) and mtROS production (Fig. 7C and D) were negated by expression of the HA-PHB2^S91A^, but not the HA-PHB2^S91D^, mutant protein. Likewise, transfection with HA-PHB2^S91A^, but not HA-PHB2^S91D^, reinstated hyperglycemia-mediated mitochondrial complex I/V inactivation in *Pgam5*-deficient cells (Fig. 7E and F). Similarly, mito-Keima reporter assays showed that, during a hyperglycemic challenge, introduction of the HA-PHB2^S91A^ mutant abrogated the rescuing effect of *Pgam5* deletion on mitophagic activity (Fig. 7G and H). Furthermore, qPCR assays revealed that HA-PHB2^S91A^ transfection blunted the positive effect of *Pgam5* deletion on mitochondrial biogenesis by reinstating transcriptional downregulation of *Pgc1α* and *Nrf2* in hyperglycemia-exposed cells (Fig. 7I and J). These results further suggest that PGAM5-mediated PHB2 dephosphorylation contributes to disruption of MQS mechanisms in hyperglycemia-challenged cardiomyocytes.

### Transgenic mice expressing a PHB2^S91D^ phosphorylation mutant gene are resistant to DCM

**Fig. 8.**
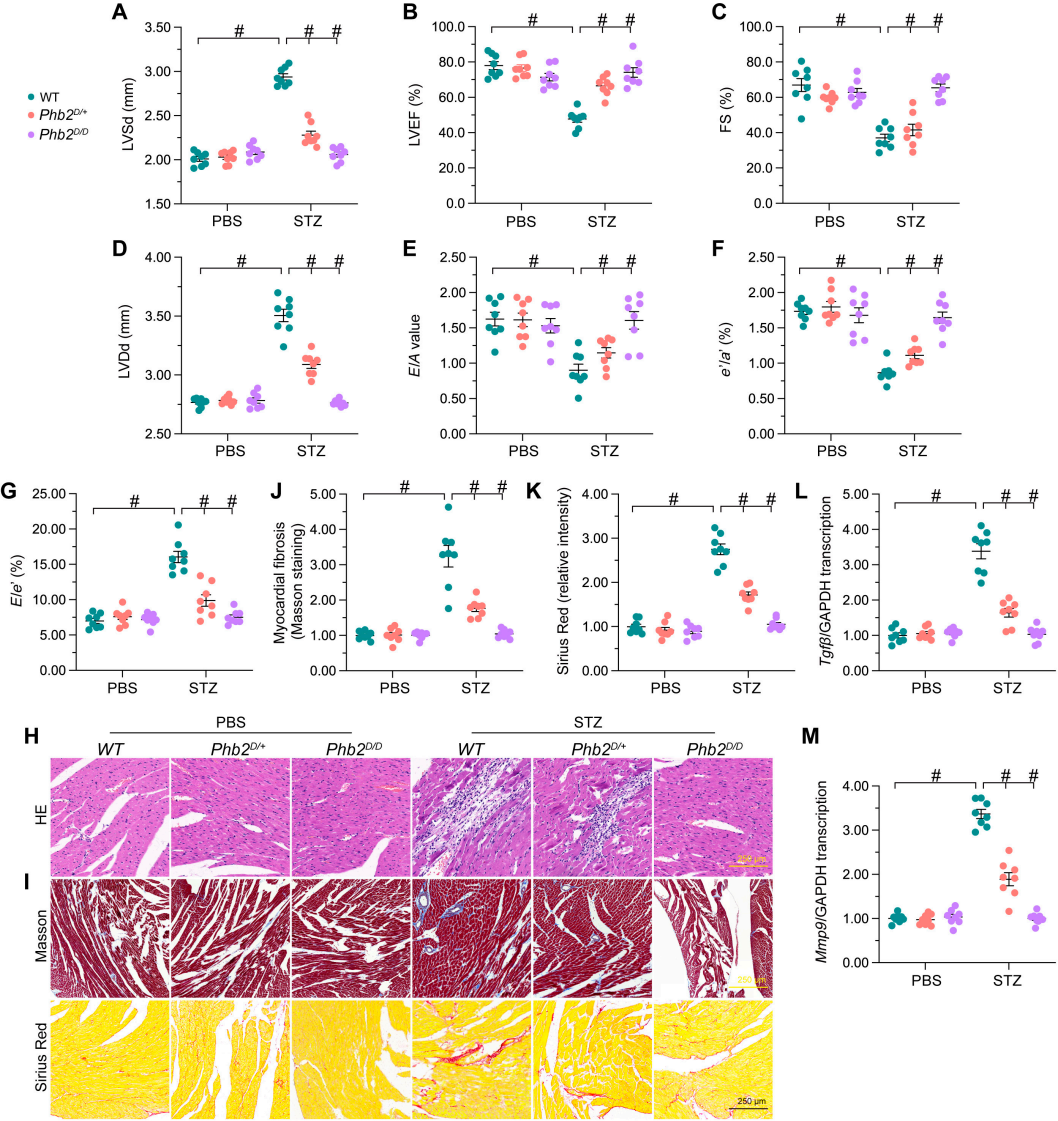
In vivo expression of a PHB2^S91^ phosphorylation mutant confers resistance to DCM. WT, heterozygous *Phb2S91^D/+^*, and homozygous *Phb2S91^D/D^* mice (*n* = 6 per type) were subjected to STZ-induced diabetes. (A to G) Echocardiography analysis. (A) LVSd, left ventricular systolic dimension; (B) LVEF, left ventricular ejection fraction; (C) FS, fractional shortening; (D) LVDd, left ventricular diastolic dimension; (E) *E*/*A*, early to late mitral flow velocities; (F) *e*′/*a*′, ratio of diastolic mitral annulus velocities; and (G) *E*/*e*′, ratio of mitral peak velocity of early filling to early diastolic mitral annular velocity. (H) Assessment of myocardial structure by H&E staining. (I) Pathological assessment of myocardial fibrosis by Masson trichrome/Sirius Red staining. (J) Quantification of Masson trichrome staining. (K) Quantification of Sirius Red staining. (L and M) Analysis of relative *Tgfβ* (L) and *Mmp9* (M) transcriptional levels in cardiac samples by qPCR. Values are presented as mean ± SEM from 6 mice per group. #*P* < 0.05.

To confirm that PHB2 dephosphorylation is a pathogenic factor in the development of DCM, we generated transgenic knockin mice carrying the *Phb2*^S91D^ variant in a C57BL/6 background. Heterozygous *Phb2S91^D/+^* or homozygous *Phb2S91^D/D^* mice grew, developed, and bred normally, possibly due to the abundant expression of p-PHB2^S91^ under normal physiological conditions (Fig. S5A and B). After STZ-induced diabetes, cardiac p-PHB2^S91^ levels were markedly downregulated in wild-type (WT) mice, partly attenuated in *Phb2S91^D/+^* mice, and unchanged in *Phb2S91^D/D^* mice (Fig. S5A and B). Diabetic *Phb2S91^D/D^* mice exhibited normal heart function (Fig. 8A to G) and myocardial structure (Fig. 8H), as assessed by echocardiography and hematoxylin and eosin (H&E) staining, respectively, compared to diabetic WT mice. Following diabetes induction, partially attenuated cardiac dysfunction (Fig. 8A to G) and moderately improved myocardial structure (Fig. 8H) were observed in *Phb2S91^D/+^* mice compared to WT mice. Meanwhile, diabetes-induced myocardial fibrosis, as evaluated by Masson trichrome staining and Sirius Red staining (Fig. 8I to K) and TGFβ/MMP9 transcription (Fig. 8I to K), were also partly attenuated in diabetic *Phb2S91^D/+^* mice and undetectable in diabetic *Phb2S91^D/D^* mice (Fig. 8L and M).

## Discussion

Mitochondrial failure is a major determinant of cardiomyocyte dysfunction and death during ischemic and metabolic disorders [32]. Utilizing gene knockout and transgenic mouse models in combination with in vitro assays in isolated cardiomyocytes, in this study, we provide evidence that PGAM5 contributes to DCM by inducing PHB2 dephosphorylation and disrupting MQS. Our study has three main findings. First, PGAM5 protein expression is upregulated and correlates with impaired heart function during STZ-induced diabetes in mice. Second, PGAM5 directly binds to IMM-localized PHB2 and causes its dephosphorylation at Ser91. Third, dephosphorylated PHB2 disturbs the MQS system in cardiomyocytes, which impairs mitochondrial dynamics, mitophagy, and mitochondrial biogenesis, contributing to myocardial dysfunction in DCM. These findings suggest that PGAM5 acts as an intracellular sensor of fluctuations in blood glucose levels and affects the phosphorylation status of PHB2 to impair mitochondrial homeostasis under hyperglycemic stress. Hence, the PGAM5–PHB2 interaction represents a novel mechanism determining MQS dysregulation, mitochondrial damage, and myocardial dysfunction in DCM. These findings provide new insights into the molecular mechanisms linking impaired MQS with compromised heart function in DCM and suggest new avenues for development of preventive and therapeutic strategies.

PGAM5 has been reported to influence progression of metabolic syndrome in multiple ways. First, PGAM5 phosphatase activity is required for activation of UCP1, a mitochondrial protein mediating adaptive thermogenesis in BAT [20]. Second, PGAM5 inhibits the production of FGF21, a regulator of the differentiation of white to brown adipocytes and of thermogenic gene expression in BAT [19]. Thus, global *Pgam5*-KO mice have reduced lipid accumulation in BAT due to increased expression of UCP1 and enhanced oxygen consumption rate [20, 33] and show both better body temperature control and an extended survival rate under combined cold stress and fasting [19]. Third, under mild stress conditions, PGAM5 serves to maintain mitochondrial integrity by promoting mitochondrial fission and mitophagy onset [34]. Interestingly, global *Pgam5*-KO mice show lean phenotypes when fed a high-fat diet, associated with decreased fat mass, enhanced glucose metabolism, reduced insulin tolerance, and augmented metabolic rate, compared to WT mice [19]. In addition, downregulation of PGAM5 in livers of high-fat diet-treated mice was associated with increased nuclear translocation and reduced mitochondrial tethering of nuclear factor erythroid-derived 2 like 2 (NFE2L2), resulting in increased expression of antioxidative gene expression [35]. Thus, a decreased capacity for fatty acid oxidation in diet-induced fatty liver disease is closely associated with PGAM5-mediated mitochondrial oxidative stress [35].

In accordance with these findings, our study suggests a critical involvement of PGAM5 in cardiomyocyte dysfunction triggered by DCM. Unlike previous studies, we used cardiomyocyte-specific, not global, *Pgam5-*KO mice, which may explain why, after STZ administration, we did not observe obvious improvement in diabetes-related end points such as fasting plasma glucose, serum cholesterol, body weight, and glucose tolerance in these animals. However, heart performance and mitochondrial function were markedly improved in diabetic *Pgam5^CKO^* mice. Taken together, our present findings and those reported previously by others suggest that PGAM5 is a core regulator in metabolic disease and is a promising candidate target for the design of novel cardioprotective drugs against DCM.

The role of PHB2 in regulating MQS has been widely explored and discussed. In heart tissue, PHB2 expression is required for mitochondrial fatty acid oxidation [23] and prevents anoxia-induced mitochondrial fission and cardiomyocyte apoptosis [36]. Our data further confirm that PHB2 is implicated in the maintenance of mitochondrial homeostasis during DCM, and this function depends on its phosphorylation status. In fact, Ross et al. [28] initially identified PHB1 and PHB2 as phosphoproteins upregulated during T cell activation. Specifically, their study showed that phosphorylation can enhance PHB2 function by promoting the formation of a PHB1/2 phosphocomplex in the IMM, a structure that proved to be critical to sustain mitochondrial function and T cell survival [28]. Although nonphosphorylated PHB2 was found to be a mitophagy receptor necessary for Parkin-related mitophagy, phosphorylation of PHB2 on Ser39 increases its affinity to LC3, suggesting that the phosphorylation status of PHB2 also regulates Parkin-independent mitophagy [25]. PHB2 phosphorylation in the cytoplasm was reported to promote PHB2 mitochondrial translocation [37]. Of note, an antiapoptotic function, possibly related to inhibition of IMM permeability, has been attributed to mitochondrial membrane-associated PHB2 expression [38]. By comparison, decreased PHB2 phosphorylation facilitates the release of proapoptotic factors from mitochondria into the cytoplasm where caspase-related mitochondrial apoptosis is activated [39]. Herein, we found that hyperglycemia had no influence on PHB2 transcription/expression but reduced the levels of p-PHB2^S91^ through a mechanism dependent on the phosphatase activity of PGAM5. Transfection of a phosphomimetic PHB2^S91D^ mutant normalized MQS, whereas a phosphodefective PHB2^S91A^ mutant abrogated the protective impact of *Pgam5* deletion. It is thus conceivable that PHB2 phosphorylation status, which is negatively modulated by PGAM5, is a critical determinant of MQS stability under diabetic conditions.

In summary, the present work suggests that diabetes-induced PGAM5 overexpression contributes to DCM through PHB2 dephosphorylation, disrupting the stabilizing effect of PHB2 on MQS in cardiomyocytes. Thus, PGAM5 and PHB2 may represent useful pharmacological targets for preventing and treating DCM.

## Materials and Methods

### Mice and DCM induction

*Pgam5^f/f^* mice were generated as previously described by us [17]. *Pgam5^CKO^* mice were generated through breeding *Pgam5^f/f^* mice to α-MHC (alpha myosin heavy chain) Cre transgenic mice (*α-MHC^Cre+^*). *Phb2S91D* knockin mice on a C57BL/6 background were generated by Cyagen Biosciences (Santa Clara, CA, USA).

DCM was induced in mice (8-week-old) through intraperitoneal injection with STZ (Sigma; 50 mg/kg dissolved in 0.1 mol/l of citrate buffer) for 5 consecutive days as previously described by us [10]. Then, 2 weeks after the last STZ injection, mice with fasting blood glucose of ≥16.7 mM were considered as diabetic and were selected for experiments. Age- and sex-matched mice treated with the same volume of citrate buffer served as nondiabetic controls.

### Echocardiography

Mice were anesthetized with 2.0% to 2.5% isoflurane, and anesthesia was maintained with 1.5% to 2.0% isoflurane. Transthoracic echocardiography was conducted using a Vevo 2100 ultrasound system (VisualSonics; Toronto, Canada). B-mode and M-mode images were acquired on short-axis orientation at the level of the midpapillary muscles. Offline measurements were conducted using the Vevo software. The same investigator performed the injections as well as echocardiography acquisition and analysis, in a blinded fashion as to the treatment groups. Treatments codes were not disclosed until all data had been analyzed.

### Biochemical analyses and mitochondrial function assessment

Mice were fasted 6 h for glucose tolerance tests (intraperitoneal glucose tolerance test, IPGTT). Glucose concentration was determined by blood isolated from the tail tip using the GE100 Blood Glucose Monitor (General Electric, Ontario, CA). Glucose solution (1 g/kg) was administered by intraperitoneal injection to *Pgam5^CKO^* mice and their littermate controls (*Pgam5^f/f^* mice). Tail blood samples were taken at designated time points. Serum cholesterol (mouse total cholesterol ELISA kit; ab285242, Abcam) and serum triglyceride (All Triglyceride Assay Kit; MBS3005147, MyBioSource, Inc.) were determined by ELISA as per the manufacturers’ instructions. Cell pellets were collected and used for ELISA analysis of mitochondrial respiratory complex I activity (ab109721, Abcam), mitochondrial respiratory complex V activity (ab109907, Abcam), and ATP production (MBS724442, MyBioSource, Inc.) according to the manufacturers’ instructions.

### Histopathological assessment and electron microscopy

Heart tissues were fixed in 10% formalin, dehydrated, and paraffin-embedded. Myocardial fibrosis was observed using a 5-μm section through Masson’s trichrome (HT15, Sigma-Aldrich). Myocardial structure was stained using hematoxylin and eosin for nuclei analysis as previously described [11]. Images were captured from similar regions on separate sections in the same biological sample using an Olympus BX51 microscope and Image-Pro Premier 9.2 (Media Cybernetics). Histopathological assessment was conducted on 4 random photomicrographs per section from ≥3 replicates per treatment. Electron microscopy was used to observe the changes of mitochondria in heart tissues as we previously described [40].

### TUNEL analysis

Analysis of apoptosis in heart sections was detected by the DeadEnd Fluorometric TUNEL System (G3250, Promega) based on the manufacturer’s instructions. Images were visualized by confocal microscopy.

### Neonatal cardiomyocyte isolation and culture

Three-day-old *Pgam5^f/f^* and *Pgam5^CKO^* mice were anesthetized with isoflurane (2%) and were killed by cervical dislocation. The hearts were mechanically dissociated, followed by enzymatic digestion with trypsin and collagenase. Then, cell suspension was resuspended after sedimentation in Dulbecco's Modified Eagle Medium (DMEM) (Gibco; 5.5 mmol/l of glucose) supplemented with 20% fetal bovine serum. The cardiomyocytes thus obtained were plated in 6-well plates (5 × 10^5^ cells/well) and cultured at 37 °C, 5% CO_2_. To induce hyperglycemic injury, cardiomyocytes were treated with high-glucose DMEM (30 mmol/l of glucose) plus 20% fetal bovine serum for 48 h.

### Cardiomyocyte contractility measurements

*Pgam5^f/f^* and *Pgam5^CKO^* mice were anesthetized with 2% isoflurane to observe the hearts that were then dissected and retrogradely perfused with Hepes–Tyrode’s buffer containing 100 mg/ml of collagenase type 2 (Worthington) via the Langendorff system. After filtration by a 100-mm mesh filter, single-cell suspension was centrifuged at 20*g* for 5 min. The pellets, which contained mostly cardiomyocytes, were washed twice with Hepes–Tyrode’s buffer containing CaCl_2_ and collected after centrifugation at 20*g*. Contractility measurements were performed in field-stimulated (1 Hz) cardiomyocytes using an IonOptix Fluorescence and Contractility System (IonOptix, MA, USA) as previously described by us [41,42]. Contractions were elicited by rectangular depolarizing pulses, 2 ms in duration and twice-diastolic threshold in intensity, using platinum electrodes. Cell shortening was measured by edge track detection, and calcium transients were measured by epifluorescence after loading the cardiomyocytes with 1 μmol/l of Fura-2 AM (#F1225, Invitrogen) for 10 min. Contractility and calcium transients were recorded, and 5 to 10 consecutive single-cell contractions during steady state were analyzed using IonWizard software (IonOptix, MA, USA).

### Western blots

Immunoblotting was conducted using total protein extracts from heart tissues or primary cardiomyocytes*.* Sodium dodecyl sulfate–polyacrylamide gel electrophoresis was used to separate the protein lysates, which was then transferred to polyvinylidene difluoride membranes. The 5% nonfat milk was applied to block the membranes, which was then incubated with primary antibodies overnight. Protein bands were detected using an ECL detection system (Pierce) after incubation with horseradish peroxidase-linked secondary antibodies. The primary antibodies used in the present study were listed in the Table S1.

### qPCR

Total RNA was isolated with the Monarch Total RNA Miniprep Kit (BioLabs, Boston, MA). Briefly, 1 μg of RNA was used for complementary DNA (cDNA) synthesis with a Superscript III reverse transcription reagent (Invitrogen, Carlsbad, CA) to assess gene expression; and 50 ng of RNA was used for cDNA synthesis with the TaqMan MicroRNA Reverse Transcription Kit (Applied Biosystems) to detect microRNA expression. PCR amplification was performed as previously described [43]. Primers for reverse transcription qPCR were designed with Primer Express software (Applied Biosystems), and relative changes in gene and microRNA expression were determined using the 2^−ΔΔCt^ method. The primers used in the present study were listed in the Table S2.

### Plasmid construction and lentiviral infection

To establish stable region-specific PHB2 and PGAM5 mutant constructs, cDNA was generated from HL-1 cells and subcloned into lenti-P2A-blast vector (generated from lenti-Cas9-blast construct, provided by GenePharma Co, Ltd., Shanghai, China). All the sequences of the constructs were validated by DNA sequencing. Empty vector without the cDNA insert was used as a negative control. The plasmids were first transfected into 293FT cells for packaging, and the virus supernatant was used to infect HL-1 cells or primary cardiomyocytes. The cells were replated 48 h later on 10-cm plates and selected using blasticidin (5 mg/ml) for 5 to 7 days to generate stable overexpression clones. Cellular transfection was conducted in mouse HL-1 cardiomyocyte cell line using Lipofectamine 3000 for 48 h. The overexpression efficiencies were finally confirmed by qPCR and western blots.

### Co-IP assay

Cardiomyocytes were lysed in IP–lysis buffer containing protease inhibitors and RNase Inhibitor (Roche, Basel, Switzerland). The supernatants were incubated with protein G-conjugated agarose (GE Healthcare Life Sciences) for 3 h, followed by centrifugation at 4 °C, 2,000*g* for 2 min to eliminate nonspecific agarose-binding proteins. Cleared supernatants were rotated overnight at 4 °C with primary antibodies and 20 μl of protein G-conjugated agarose was used to precipitate protein–antibody mixtures for 4 h. The precipitates were then washed 3 times and detected via western blotting.

### Mitochondrial ROS and mPTP opening detection

To measure mtROS levels, cells were washed three times with phosphate-buffered saline (PBS) and loaded with 2 μM of the fluorescent mitochondrial superoxide indicator MitoSOX Red (M36008, Invitrogen) for 30 min at 37 °C. Cells were then observed using an Olympus IX73 microscope, and signals were quantified using cellSens software. To track mPTP opening, primary cardiomyocytes were loaded with TMRE as described in our previous studies [42,44]. TMRE fluorescence was measured using a Nikon confocal microscope system.

### ΔΨm and mitophagy detection

Variations in ΔΨm were recorded in primary cardiomyocytes stained using JC-1 (T3168, Invitrogen). Mitophagic activity was assessed by transfection of a mito-Keima reporter (a mitochondrially localized pH-sensitive protein) by calculating the cellular area occupied by puncta with a high 561/457 nm ratio, indicating the fusion of mitochondria in lysosomes.

### Statistical analysis

Statistical Package for the Social Sciences (SPSS) software (version 18) was used to perform the statistical analysis, and data were presented as mean ± standard error of the mean (SEM). Comparison among multiple groups (genotype × treatment) was performed by two-way analysis of variance followed by Bonferroni post hoc test. All in vitro experiments were done in triplicate and performed independently 2 to 5 times. Each *n* value corresponds to an independent experiment. For in vivo data, each *n* value corresponds to an individual mouse, and the number of mice used for each in vivo analysis is indicated in the figure legends. Statistical significance was recognized at *P* < 0.05.

## Data Availability

All data generated or analyzed during this study are included in this published article.

## Supplementary Materials

Supplementary Materials Fig. S1. Cardiac PGAM5 expression is upregulated by hyperglycemia and contributes to metabolic disorder. Fig. S2. PGAM5 deletion normalizes MQS in cardiomyocytes exposed to hyperglycemia. Fig. S3. PGAM5 has no influence on PHB2 transcription and expression. Fig. S4. PGAM5 binds and dephosphorylates PHB2. Fig. S5. In vivo expression of a PHB2^S91^ phosphorylation mutant confers resistance to DCM. Table S1. Antibody information in western blot. Table S2. Primers for qPCR.
