# Erratum to “PGAM5-Mediated PHB2 Dephosphorylation Contributes to Diabetic Cardiomyopathy by Disrupting Mitochondrial Quality Surveillance”

**DOI:** 10.34133/research.0325

**Published:** 2024-02-29

**Authors:** Rongjun Zou, Jun Tao, Jie He, Chaojie Wang, Songtao Tan, Yu Xia, Xing Chang, Ruibing Li, Ge Wang, Hao Zhou, Xiaoping Fan

**Affiliations:** ^1^ Department of Cardiovascular Surgery, Guangdong Provincial Hospital of Chinese Medicine, the Second Affiliated Hospital of Guangzhou University of Chinese Medicine, Guangzhou 510120, Guangdong, China.; ^2^ The Second Clinical College of Guangzhou University of Chinese Medicine, Guangzhou 510405, Guangdong, China.; ^3^ Department of Cardiovascular Surgery, Sun Yat-sen Memorial Hospital, Sun Yat-sen University, Guangzhou 510120, Guangdong, China.; ^4^ Senior Department of Cardiology, The Sixth Medical Center of People’s Liberation Army General Hospital, Beijing 100048, China.; ^5^ Guang’anmen Hospital, China Academy of Chinese Medical Sciences, Beijing 100053, China.

In the Research Article “PGAM5-mediated PHB2 dephosphorylation contributes to diabetic cardiomyopathy by disrupting mitochondrial quality surveillance” [1], an inadvertent error was discovered where one image was mistakenly included in Fig. 7H. Specifically, during the figure assembly process, an uncorrected picture of the Pgam5f/f+HG+PHB2S91A group was mistakenly presented in Fig. 7H. The authors want to assure readers that this issue has been promptly addressed, and the corrected image of the Pgam5f/f+HG+PHB2S91A group for Fig. 7H is below. Importantly, it should be noted that this error does not affect the scientific conclusions drawn in the study. The authors sincerely apologize for any inconvenience caused by this oversight.

**Fig. 7.**
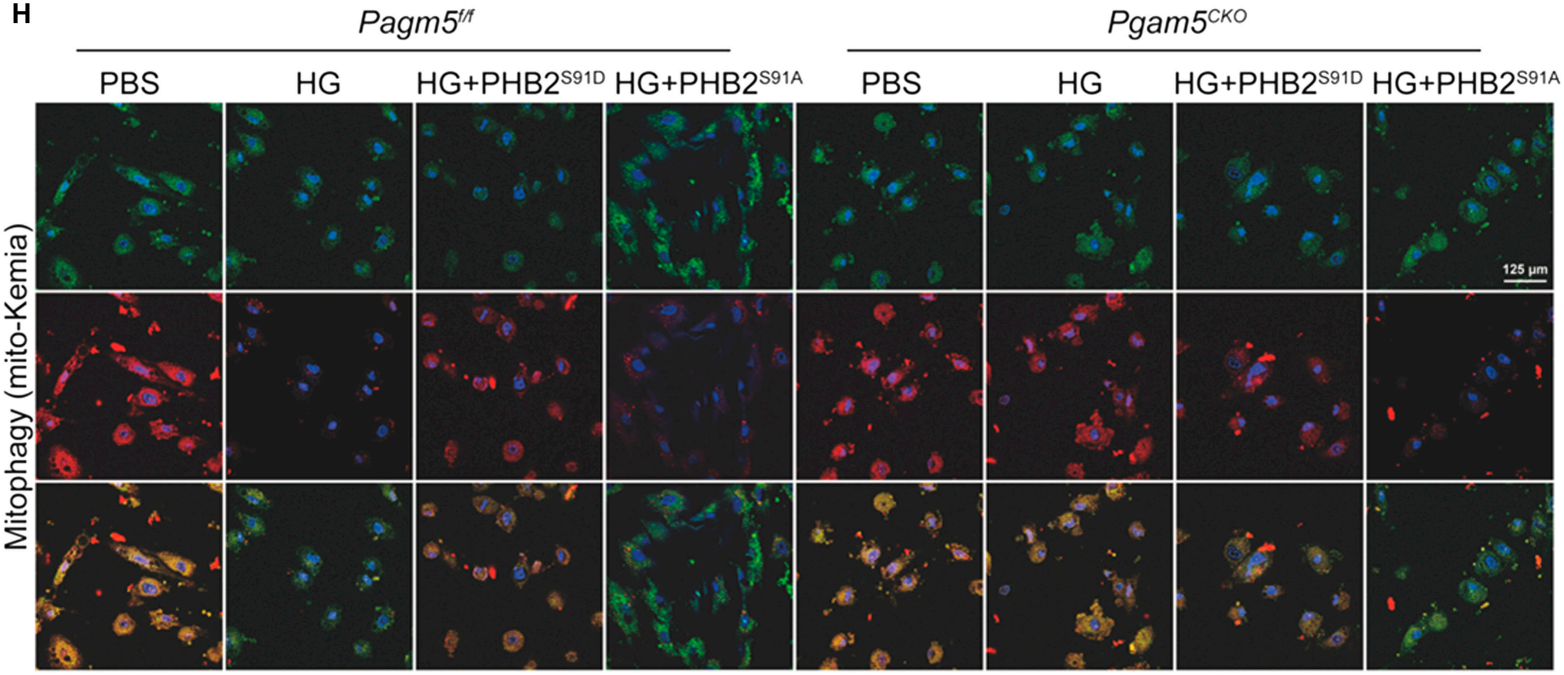
Presentative pictures of cardiomyocyte transfected with mito-Keima.
