# Supplementary material for: PGAM5-Mediated PHB2 Dephosphorylation Contributes to Diabetic Cardiomyopathy by Disrupting Mitochondrial Quality Surveillance
Source: Research (Wash D C). 2022 Dec 15;2022:0001. doi: 10.34133/research.0001 (PMC11404314; doi:10.34133/research.0001)
Supplement: Supplementary Materials — Fig. S1. Cardiac PGAM5 expression is upregulated by hyperglycemia and contributes to metabolic disorder. Fig. S2. PGAM5 deletion normalizes MQS in cardiomyocytes exposed to hyperglycemia. Fig. S3. PGAM5 has no influence on PHB2 transcription and expression. Fig. S4. PGAM5 binds and dephosphorylates PHB2. Fig. S5. In vivo expression of a PHB2S91 phosphorylation mutant confers resistance to DCM. Table S1. Antibody information in western blot. Table S2. Primers for qPCR. [file 0001.f1.docx]

Supplemental figures and tables for

**PGAM5-mediated PHB2 dephosphorylation contributes to diabetic cardiomyopathy by disrupting mitochondrial quality surveillance**

Rongjun Zou^1,2#^, Jun Tao^3#^, Jie He^1,2#^, Chaojie Wang^1,2^, Songtao Tan^1,2^, Yu Xia^1,2^, Xing Chang^4,5^, Ruibing Li^4^, Ge Wang^1,2*^, Hao Zhou^4,5*^, Xiaoping Fan^1,2*^

^1^Department of Cardiovascular Surgery, Guangdong Provincial Hospital of Chinese Medicine, the Second Affiliated Hospital of Guangzhou University of Chinese Medicine, Guangzhou 510120, Guangdong, China

^2^The Second Clinical College of Guangzhou University of Chinese Medicine, Guangzhou 510405, Guangdong, China

^3^Department of Cardiovascular Surgery, Sun Yat-sen Memorial Hospital, Sun Yat-sen University, Guangzhou, China 510120, Guangdong, China

^4^Senior Department of Cardiology, The Sixth Medical Center of People’s Liberation Army General Hospital, Beijing 100048 Beijing, China

^5^Guang’anmen Hospital, China Academy of Chinese Medical Sciences, Beijing 100053, China

#The first three authors contributed to this article equally.

*Corresponding Authors

Ge Wang, email: gwo.wong@icloud.com

Department of Cardiovascular Surgery, Guangdong Provincial Hospital of Chinese Medicine, the Second Affiliated Hospital of Guangzhou University of Chinese Medicine, Guangzhou 510120, Guangdong, China

Hao Zhou, email: zhouhao@plagh.org

Senior Department of Cardiology, The Sixth Medical Center of People’s Liberation Army General Hospital, Beijing 100048 Beijing, China

Xiaoping Fan, email: fukui-hanson@hotmail.com

Department of Cardiovascular Surgery, Guangdong Provincial Hospital of Chinese Medicine, the Second Affiliated Hospital of Guangzhou University of Chinese Medicine, Guangzhou 510120, Guangdong, China

**Supplemental Figures**

**
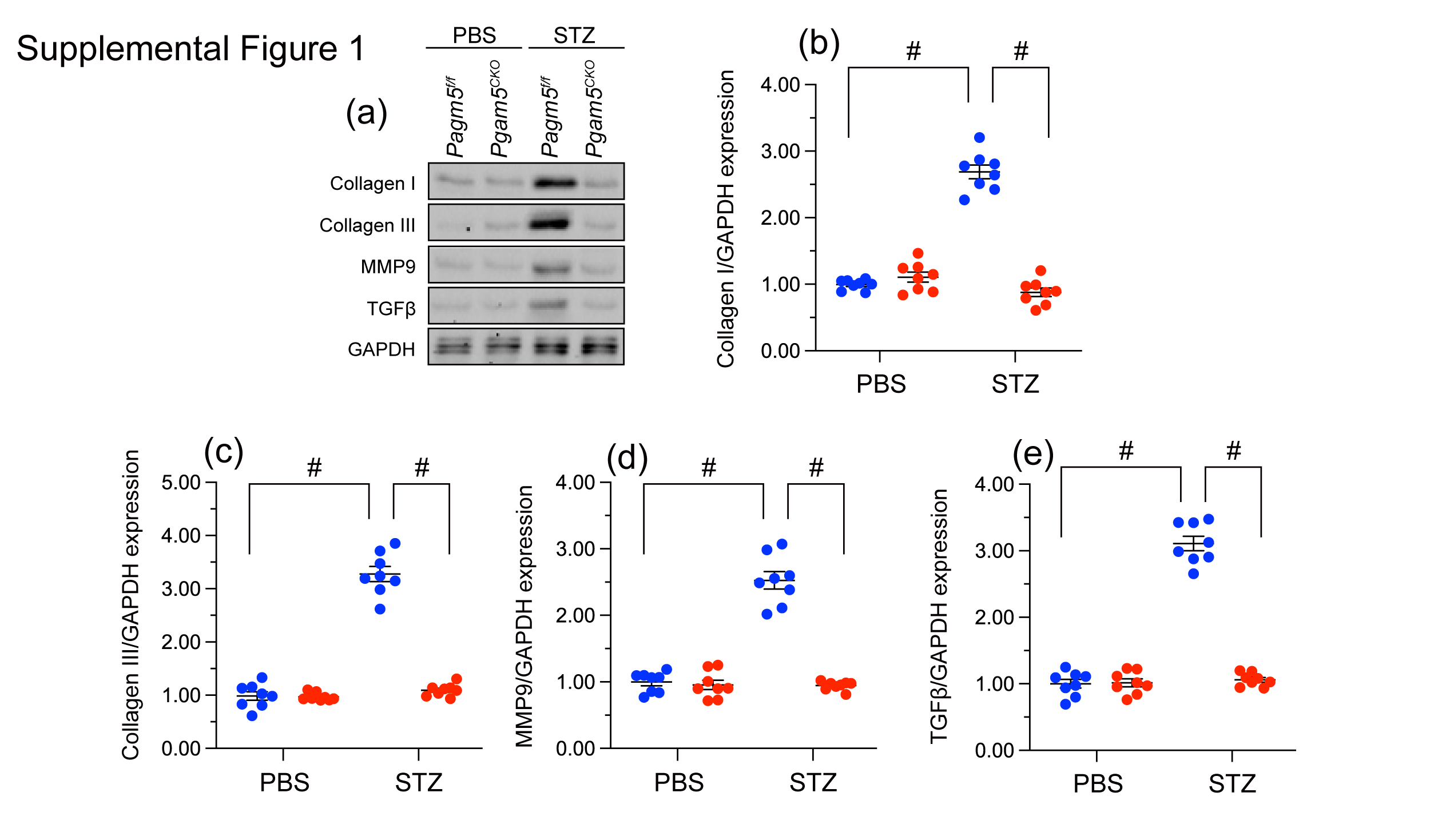
**

**Figure S1. Cardiac PGAM5 expression is upregulated by hyperglycemia and contributes to metabolic disorder.** *In vivo*, the cardiomyocyte-specific *Pgam5* knockout (*Pgam5^CKO^*) and *Pgam5^f/f^* mice were injected intraperitoneally with STZ (50mg/Kg dissolved in 0.1mol/L citrate buffer) for five consecutive days to induce diabetes. Non-diabetic mice were the age- and sex-matched, which injected with the same volume PBS. **(a-e)** Western blot analysis of collagen I, collagen III, TGFβ, and MMP9 expression in cardiac tissue. Values are presented as mean ± SEM; For biochemical determinations, n = 6 mice per group. P<0.05.

**
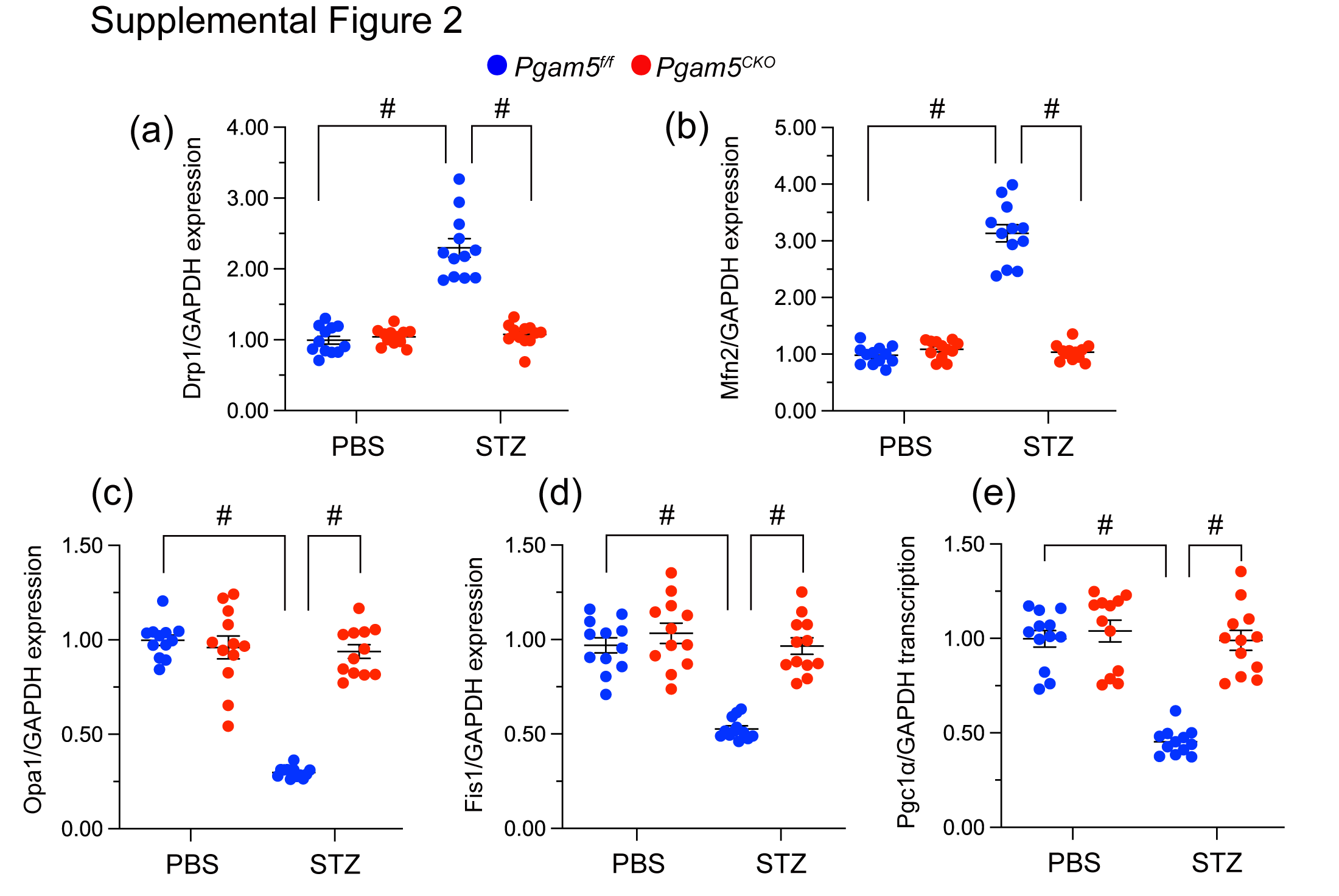
**

**Figure S2. PGAM5 deletion normalizes MQS in cardiomyocytes exposed to hyperglycemia.** *In vivo*, the cardiomyocyte-specific *Pgam5* knockout (*Pgam5^CKO^*) and *Pgam5^f/f^* mice were injected intraperitoneally with STZ (50mg/Kg dissolved in 0.1mol/L citrate buffer) for five consecutive days to induce diabetes. Non-diabetic mice were the age- and sex-matched, which injected with the same volume PBS. **(a-e)** qPCR analysis of relative expression of *Drp1*, *Fis1*, *Mfn2*, *Opa1*, and *Pgc1α* in cardiac tissue. Values are presented as mean ± SEM. For *in vivo* data, n = 6 mice per group. P<0.05.

**
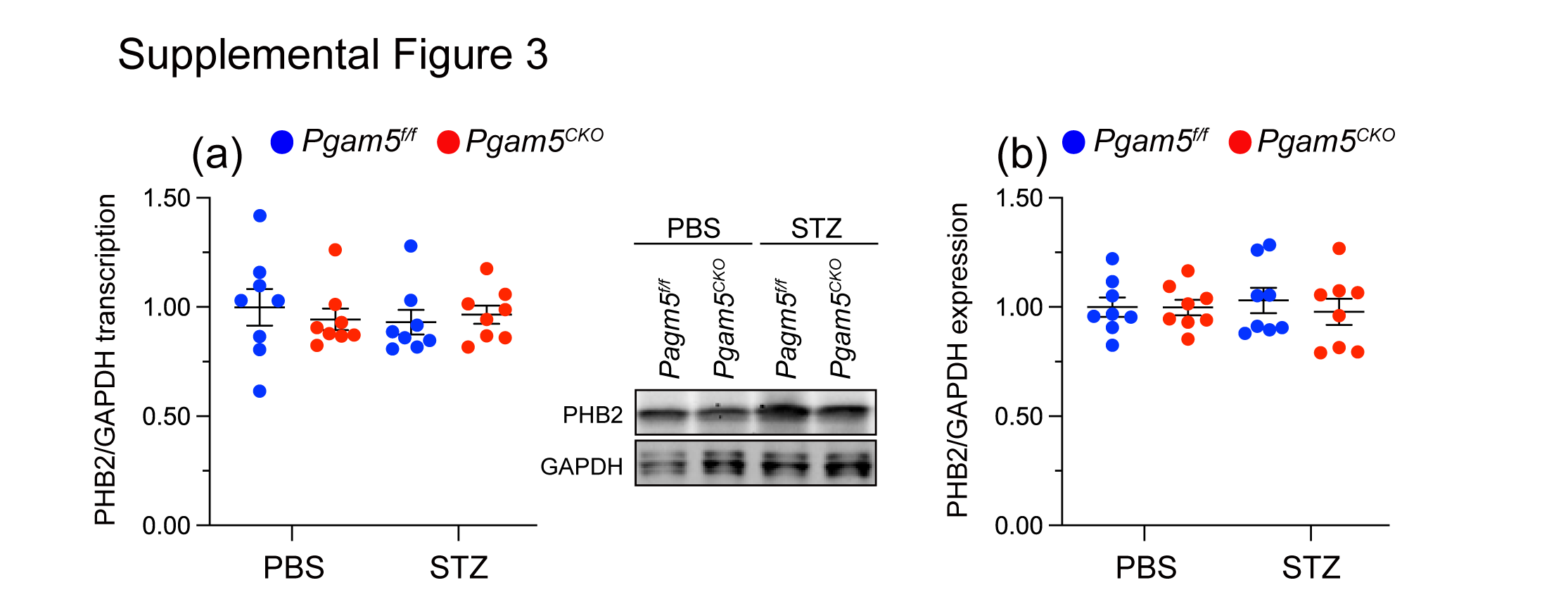
**

**Figure S3. PGAM5 has no influence on PHB2 transcription and expression.** Mouse HL-1 cardiomyocyte cell line were cultured under high-glucose medium (30 mmol/L glucose) for 48 hrs to induce hyperglycemia damage *in vitro*. HL-1 cells treated with normal-glucose medium (5.5 mmol/L glucose) was used as the control group. **(a)** Analysis of relative transcription levels of *Phb2* in cardiac tissues by qPCR. **(b)** Western blot analysis of PHB2 expression in heart tissues. Values are presented as mean ± SEM. For *in vivo* data, n = 6 mice per group. P<0.05.

**
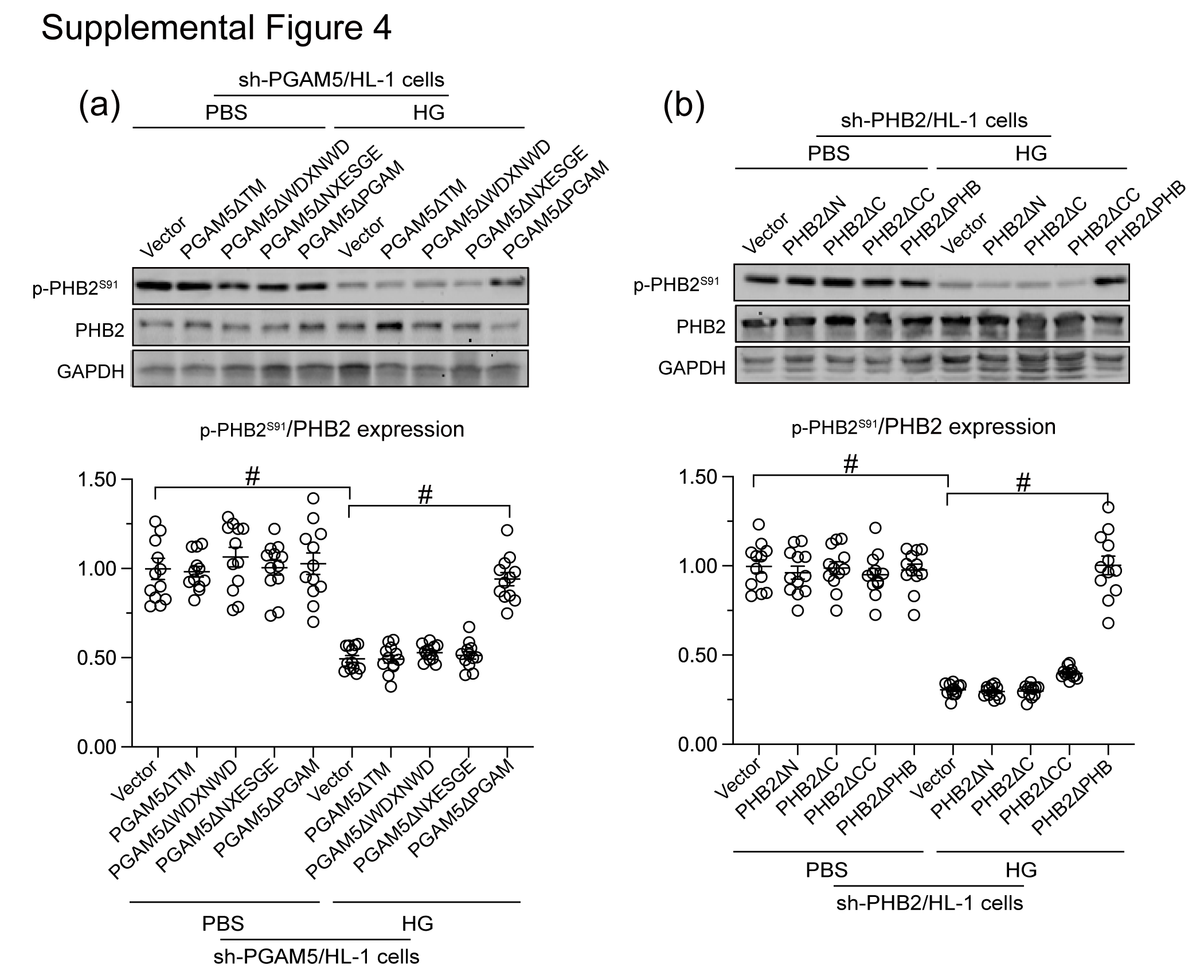
**

**Figure S4. PGAM5 binds and dephosphorylates PHB2.** Mouse HL-1 cardiomyocyte cell line were cultured under high-glucose medium (30 mmol/L glucose) for 48 hrs to induce hyperglycemia damage *in vitro*. HL-1 cells treated with normal-glucose medium (5.5 mmol/L glucose) was used as the control group. **(a)** Western blot analysis of p-PHB2^S91^ expression in HL-1 cells transfected with region-specific PGAM5 mutants. The expression of p-PHB2^S91^ was normalized to that of total PHB2. **(b)** Western blot analysis of p-PHB2^S91^ in HL-1 cells transfected with region-specific PHB2 mutants. The expression of p-PHB2^S91^ was normalized to that of total PHB2. Values are presented as mean ± SEM. For *in vivo* data, n = 6 mice per group. For *in vitro* data, n = 4 independent experiments. P<0.05.

**
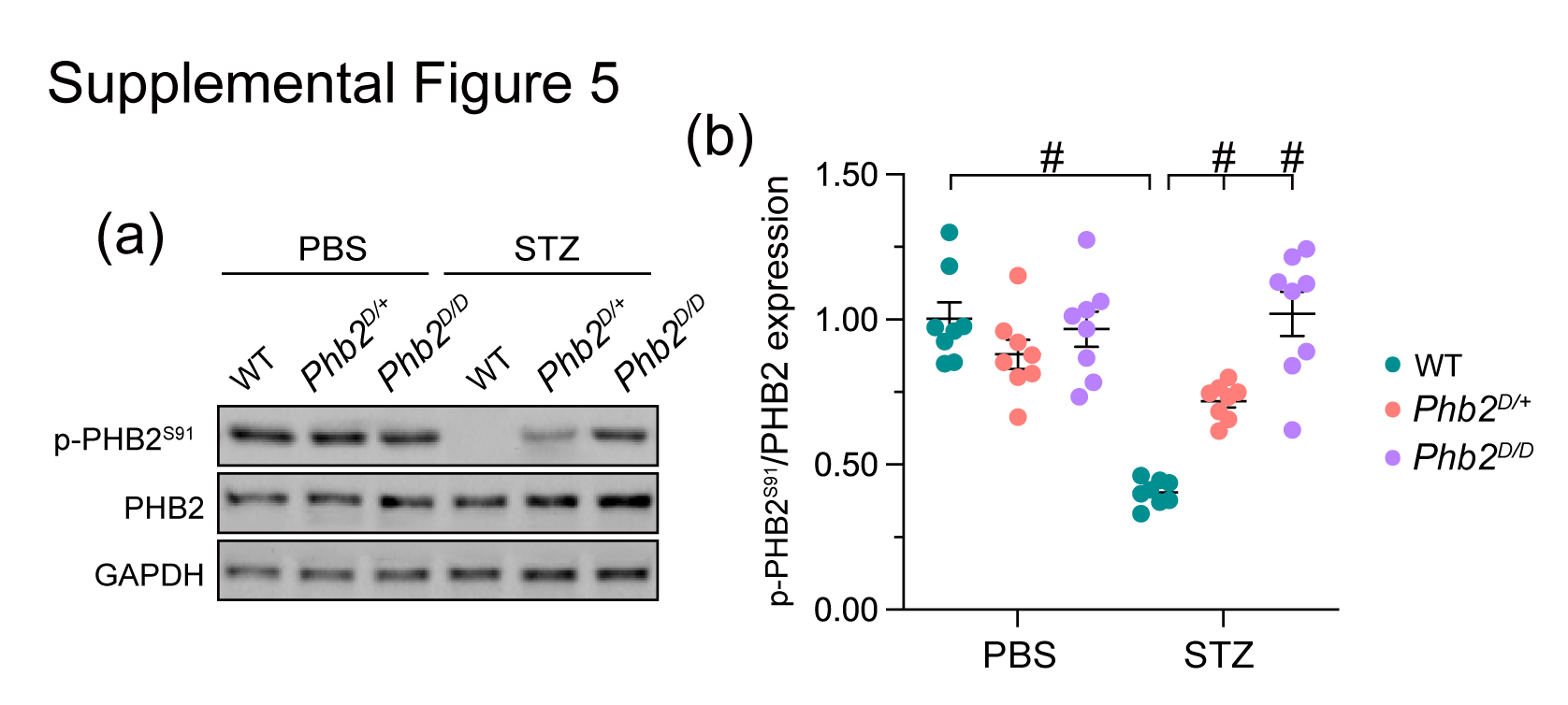
**

**Figure S5. *In vivo* expression of a PHB2^S91^ phosphorylation mutant confers resistance to DCM.** WT, heterozygous *Phb2S91^D/+^*, and homozygous *Phb2S91^D/D^* mice (n = 6 per type) were subjected to STZ-induced diabetes. **(a, b)** Western blots analysis of p-PHB2^S91^ expression in cardiac samples. Data were normalized to total PHB2 expression. Values are presented as mean ± SEM from 6 mice per group. P<0.05.

**Table S1: Antibody information in Western blot**

| Name | Catalogue number | Dilution factor |
| --- | --- | --- |
| Drp1 | Abcam, #ab184247 | 1:1000 |
| Fis1 | Abcam, #ab156865 | 1:1000 |
| Mfn2 | Abcam, #ab124773 | 1:1000 |
| Opa1 | Abcam, #ab42364 | 1:1000 |
| GAPDH | Abcam, #ab8245 | 1:1000 |
| Tom20 | Abcam, #ab186735 | 1:1000 |
| Collagen I | Abcam, #260043 | 1:1000 |
| Collagen III | Abcam, #7778 | 1:1000 |
| TGFβ | Abcam, #215715 | 1:1000 |
| MMP-9 | Abcam, #228402 | 1:1000 |
| PGAM5 | MyBioSource, #MBS9612114 | 1:1000 |
| PHB2 | Cell Signaling Technology, #14085 | 1:1000 |

**Table S2: Primers for qPCR**

| Gene | Forward Prime | Reverse Prime |  |
| --- | --- | --- | --- |
| *Pgc1α* | 5′-CGGAAATCATATCCAACCAG-3′ | 5′-TGAGGACCGCTAGCAAGTTTG-3′ | |
| *Nrf2* | 5′-CCTCGCTGGAAAAAGAAGTG-3′ | 5′-GGAGAGGATGCTGCTGAAAG-3′ | |
| *Tfam* | 5′-GGCGAATTCCTCGAGGCCACCATG  GCGCTGTTCCGGGGAATGT-3′ | 5′- CATACGCGTATGCTCAGAGATGTC  TCCGGATCGT -3′ | |
| *Pgam5* | 5′-GAACTACATCCACCGAGCTGA-3′ | 5′-GGGAAACTGCAACGCTCTAC-3′ | |
| *Tgfβ* | 5′-TACCATGCCAACTTCTGTCTGGGA-3′ | 5′-ATGTTGGACAACTGCTCCACCTTG-3′ | |
| *Gapdh* | 5′-ACGGCAAATTCAACGGCACAGTCA-3′ | 5′-TGGGGGCATCGGCAGAAGG-3′ | |
| *Mmp9* | 5′-CCATCGATTAGAAGCAGGAGGACCCGA-3′ | 5′-GGACTAGTTGGCTAACGCTGCCTTTG-3′ | |
| *Phb2* | 5′-AGCAGGAACAGCACAGAAGA-3′ | 5′-CGGAGCTTGATATAGCCAGGAT-3′ | |
